# Understanding the Relationship Between the Multidimensional Perfectionism and Self-Compassion in Adults: The Effect of Age

**DOI:** 10.5964/ejop.11981

**Published:** 2023-11-30

**Authors:** Athena Daniilidou

**Affiliations:** 1 Department of Educational and Social Policy, University of Macedonia, Thessaloniki, Greece; Università Cattolica del Sacro Cuore, Milan, Italy

**Keywords:** adulthood, age, perfectionism, self-compassion

## Abstract

Literature suggests that perfectionism is associated to self-compassion. However, the multiple relationships between the types of perfectionism (adaptive, maladaptive and non-perfectionists) and the multidimensional construct of self-compassion have not been thoroughly examined. To this end, the present study aimed (a) to examine the relationships between the types of perfectionism and the self-compassion components in an adult sample and (b) to check the effect of age on the relationship between the perfectionistic types and the self-compassion components. Participants were 509 adults aged 18 to 65 years. Self-report questionnaires were used to collect the data. Results indicated that High Standards positively predicted all self-compassion components while Discrepancy positively predicted Self-judgment and Isolation and overidentification and negatively predicted Self-Kindness and mindfulness and Common humanity. In addition, it was found that adaptive perfectionists and non-perfectionists reported higher levels on the positive components of self-compassion and lower levels on its negative components, compared to maladaptive perfectionists. With respect to age, participants in established (30–45 years) and middle (46–65 years) adulthood reported higher levels on the positive self-compassion components and lower levels on its negative components compared to young adults (18–29 years), while participants in emerging adulthood scored higher on both the dimensions of perfectionism (adaptive and maladaptive) compared to participants in established and middle adulthood. Finally, age moderated only the relationship between adaptive perfectionism and Isolation and overidentification. Future directions and implications are being discussed.

Research so far suggests that perfectionism is often associated with maladaptive behaviors and negative outcomes (Limburg et al., 2017; Smith et al., 2018) and interferes and/or possibly disrupts the development of self-compassion (Linnett & Kibowski, 2020), while self-compassion has been linked to several positive behaviors and well-being (Biber & Ellis, 2019; Braun et al., 2016). However, it still remains unclear how perfectionism and self-compassion are interrelated, when the types of perfectionism (adaptive, maladaptive, non-perfectionism) are involved. More specifically, although studies have found that maladaptive perfectionism is negatively associated to overall self-compassion and/or its positive components (e.g., Linnett & Kibowski, 2020; Yeshua et al., 2019), the multiple relationships between the types of perfectionism and the positive and negative self-compassion components have not been thoroughly examined. According to Linnett and Kibowski (2020), self-compassion is a multidimensional construct, a fact overlooked by some studies that examine the relationships between the two constructs by assessing self-compassion as a unidimensional construct (e.g., Ferrari et al., 2018; Mehr & Adams, 2016). Thus, so far, the different associations between the perfectionistic types and the self-compassion components have not been sufficiently conceptualized in the literature.

To this end, the present study was designed to explore the relationships between multidimensional perfectionism and the components of self-compassion in adults, and to determine if the relation between the types of perfectionism and the self-compassion components could be moderated by participants’ age.

## Defining Self-Compassion

Although self-compassion is a relatively new concept in the international literature, since Neff (2003a, 2003b) published the first studies on the topic, researchers’ interest on the concept has been increased. The construct of self-compassion has been conceptualized by Neff (2003b) as a way of relating to ourselves during hardships, predicaments, and difficult thoughts and/or feelings. In other words, self-compassion requires from the individuals to be open on their suffering, not avoiding it, offering a nonjudgmental understanding to their pain (Neff, 2011). However, having self-compassion does not mean that one cannot identify their failures and/or inadequacies. Rather, a compassionate attitude towards oneself involves being patient and kind towards negative feelings and requires a balanced perspective (Bennett-Goleman, 2001).

According to Neff's (2003b) definition, self-compassion entails six interconnected components that represent positive and negative poles of three dimensions: Self-Kindness versus self-judgment, feelings of common humanity versus isolation, and mindfulness versus over-identification. The first dimension is both a trait and a psychological process and refers to the tendency of being gentle and comforting ourselves rather than criticizing and judging our mistakes, failures, or personal inadequacies. Feelings of common humanity involve the acknowledgment of the vulnerable human nature. There are certain emotions that all people experience during challenging circumstances and this inclusive perspective helps connect with each other. Finally, mindfulness refers to being aware of the momentary experience in an equilibrated way that involves taking a meta-perspective on how one feels with equanimity rather than exaggeration.

Studies on the relationship between self-compassion and age suggest that there is a small but significant association between them, with self-compassion increasing as the individual gets older (Homan, 2016; Neff, 2023; Neff & Pommier, 2013). Lee et al. (2021) examined self-compassion in a large community sample and the results showed that self-compassion levels peaked around the age of 77 years. Murn and Steele (2020) studied the age-related changes in self-compassion and its components (mindfulness, sense of isolation or connectedness with others, self-kindness towards oneself, common humanity) and noticeable shifts that occur over time were highlighted. Specifically, an increase in mindfulness among older adults compared to younger ones was found, while younger individuals scored lower on common humanity and mindfulness compared to older participants. Similarly, Bratt and Fagerström (2020) found that increased age was associated with lower scores on the self-criticism component of self-compassion and higher scores on the common humanity component. Studies in Greece showed that older adults (50–72 years) demonstrated higher levels of the positive and lower levels of the negative self-compassion components compared to younger age groups (18–30 and 31–49 years) (Karakasidou et al., 2020). It may be that the accumulated knowledge that comes with maturity and life experiences allows for a kinder and more compassionate attitude towards oneself (Neff, 2023). However, there is also evidence suggesting that there is no relationship between self-compassion and age (Benzo et al., 2017; Neff & McGehee, 2010; Phillips & Ferguson, 2013).

## Defining Perfectionism

Perfectionism is a personality disposition that has been described as the need of the individuals “to be perfect in all aspects of their lives” (Flett & Hewitt, 2002, p. 5). Perfectionists strive for success, have high standards and experience the internal urge to display perfection and be flawless (Flett & Hewitt, 2002). In the relevant literature, there are two different perspectives in defining perfectionism. Some researchers consider perfectionism as a negative trait, while others suggest that the construct is multidimensional and they have established a distinction between the positive/adaptive and the negative/maladaptive dimension of perfectionism (e.g., Burns, 1980; Hewitt et al., 2003; Stoeber & Otto, 2006). According to Stoeber & Otto (2006), adaptive and maladaptive perfectionists can be distinguished by their perfectionistic strivings and their perfectionistic concerns, two dimensions which correspond to the positive and negative aspect of perfectionism respectively. Individuals with high levels of perfectionistic strivings and low levels of perfectionistic concerns can be conceived as healthy perfectionists, individuals with high levels of perfectionistic strivings and high levels of perfectionistic concerns as unhealthy perfectionists, and individuals with low levels of perfectionistic strivings as non-perfectionists (Stoeber & Otto, 2006).

In the same direction, Slaney and his colleagues (2002) argued that perfectionism has both adaptive and maladaptive aspects. In their approach, adaptive and maladaptive perfectionists can be distinguished by taking into consideration two dimensions: High Standards and Discrepancy. High Standards refer to the high personal standards one sets for oneself and Discrepancy describes the difference between the standards one has for oneself and the evaluation of their actual performance (Slaney et al., 2001). Researchers suggest that discrepancy is an indicator of perfectionistic concerns and, thus, it describes maladaptive perfectionism (Suddarth & Slaney, 2001). On the other hand, the high standards dimension is an indicator of adaptive perfectionism, but it can appear as maladaptive when discrepancy is high (Rice & Slaney, 2002). Thus, individuals with low high standards represent the type of non-perfectionists, individuals with high discrepancy and high standards are described as maladaptive perfectionists and those who have high standards but low discrepancy represent the adaptive type of perfectionists.

Studies examining the relationship between perfectionism and age present inconsistent findings (Robinson et al., 2021). Some studies have concluded that there is a decrease in perfectionism with age (e.g., Landa & Bybee, 2007; Sastre-Riba et al., 2016), while others have not found evidence of association between perfectionism and age (Diamantopoulou & Platsidou, 2014; Schweitzer & Hamilton, 2002). However, it must be noted that most studies in the relevant literature mainly refer to young adults while middle-aged and older adults (especially over 60) have been overlooked (Robinson et al., 2021). In addition, the vast majority of the studies tend to examine age differences in terms of overall perfectionism (e.g., Sand et al., 2021; Schweitzer & Hamilton, 2002) or perfectionism dimensions (perfectionistic strivings and concerns) (e.g., Sastre-Riba et al., 2016) rather than the individual perfectionistic types such as adaptive, maladaptive and non-perfectionists.

## The Relationship Between Self-Compassion and Perfectionism

Literature suggests that maladaptive perfectionism dimensions, such as concern over mistakes and discrepancy, are negatively related to and predict overall self-compassion and/or its positive components (i.e., mindfulness, self-kindness, common humanity) and positively related to and predict its negative components (i.e., isolation and over-identification, self-judgment) (e.g., Linnett & Kibowski, 2020; Stoeber, Madigan, & Gonidis, 2020; Yeshua et al., 2019). These findings are not surprising given that maladaptive perfectionistic attitudes are characterized by excessive self-criticism and struggle to maintain a positive and non-judgmental self-attitude compared to self-compassionate attitudes which are characterized by being tolerant over flaws and inadequacies (Linnett & Kibowski, 2020).

On the other hand, findings on the association between the adaptive perfectionism dimensions and self-compassion are mixed. Some studies found that the adaptive perfectionism dimensions such as perfectionistic strivings have been associated with and predict lower overall self-compassion (e.g., Hiçdurmaz & Aydin, 2017; Mosewich et al., 2011). Other researchers found that the adaptive perfectionism dimensions such us striving for excellence are not associated with overall self-compassion and only weakly positively associated with and predict the negative self-compassion component namely self-judgment (Linnett & Kibowski, 2020). Finally, there is also evidence that perfectionistic strivings are not associated with or predict a change in self-compassion levels (e.g., Neff, 2003a).

To summarize, both perfectionistic concerns and perfectionistic strivings have been found to predict lower levels of overall self-compassion although the predictive effect of perfectionistic strivings was found smaller compared to the effect of perfectionistic concerns (e.g., Hiçdurmaz & Aydin, 2017; Mosewich et al., 2011). However, although research findings consistently suggest that the maladaptive dimension of perfectionism negatively predicts overall self-compassion and/or its positive components, further research on the predictive role of the adaptive perfectionism dimension on the self-compassion components is required along with the fact that findings concerning the association between the perfectionistic types (adaptive, maladaptive, non-perfectionists) and the positive and negative self-compassion components are even more limited.

## Aims and Hypotheses of the Study

Taking the above into consideration, the present study was designed to fill this gap and to broaden our understanding on how the different perfectionistic types (adaptive, maladaptive, non-perfectionists) are associated with the positive and negative self-compassion components. In addition, findings concerning the effect of age on the relationship between the aforementioned constructs are rather limited, especially for adults and older adults. To this end, this study aims (a) to examine the relationships between the types of perfectionism and the self-compassion components in an adult sample and (b) to check the effect of age on the relationship between the perfectionistic types (adaptive, maladaptive, non-perfectionists) and the positive and negative self-compassion components.

Based on the literature summarized above, it was first hypothesized that the maladaptive perfectionism dimension (Discrepancy) will negatively predict the positive self-compassion components and positively predict the negative self-compassion components (*Hypothesis 1a*) and the adaptive perfectionism dimension (High Standards) will probably not predict any of the self-compassion components (*Hypothesis 1b*). In addition, based on the relevant research findings it was expected that the perfectionistic types would present differences on self-compassion components. More specifically, it was hypothesized that non-perfectionists and adaptive perfectionists will report higher levels on the positive components of self-compassion and lower levels on the negative components of self-compassion (*Hypothesis 2a*), while maladaptive perfectionists will report higher levels on the negative components of self-compassion and lower levels on the positive components of self-compassion (*Hypothesis 2b*). Finally, based on the prior research findings indicating that both perfectionism and self-compassion vary with proceeding age, it was hypothesized that participants will present differences in the perfectionism dimensions and self-compassion components in relation to their age (*Hypothesis 3a*) and that age will moderate the relationship between the perfectionistic types and the self-compassion components (*Hypothesis 3b*).

## Method

### Participants

Data were collected from 509 adults from urban and suburban areas in Greece. The sample consisted of 317 women (62.3%) and 192 men (37.7%). Their age ranged from 18 to 65 years, with an average age of 44.42 years (*SD* = 15.55). The majority (71.3%) were married (*N* = 301) or in a stable relationship (*N* = 62). With regards to their educational status, 62 participants had completed high school (12.2%), 26 were college students (5.1%), 277 held a Bachelor’s Degree (54.4%), 126 held a Master’s Degree (24.8%) and 18 (3.5%) a Doctoral Degree.

### Materials

#### Self-Compassion Scale (SCS)

The Self-Compassion Scale (SCS)—designed by Neff (2003a) and translated into Greek by Mantzios and his colleagues (Mantzios et al., 2015)—was used to assess self-compassion. It consists of 26 items which assess six components of self-compassion: Self-Kindness (5 items, e.g., *I try to be loving towards myself when I’m feeling emotional pain*), Self-judgement (5 items, e.g., *I’m intolerant and impatient towards those aspects of my personality I don't like*), Common humanity (4 items, e.g., *I try to see my failings as part of the human condition*), Isolation (4 items, e.g., *When I’m feeling down, I tend to feel like most other people are probably happier than I am*), Mindfulness (4 items, e.g., *When something upsets me I try to keep my emotions in balance*) and Overidentification (4 items, e.g., *When I’m feeling down I tend to obsess and fixate on everything that’s wrong*). Respondents answer each item on a 5-point Likert-type scale ranging from 1 (almost never) to 5 (almost always). Subscale scores are computed by calculating the mean of subscale item responses. A total score indicating the total self-compassion of the individual can also be extracted, after reversing the values in the items with the negative meaning (self-judgment, isolation, and over-identification) (Neff, 2003a).

In the present study, an exploratory factor analysis of the SCS proceeded which revealed a four-factor solution that partially matched the inner structure of the original scale. Seven items were removed from the scale since they did not load on the factors proposed by the constructors and the analysis was repeated in the 19 remaining variables. In the final model, four factors were abstracted explaining the 53.76% of the total variance: Self-Kindness and mindfulness (Items 5, 9, 12, 17, 19, 22, 26, Eigenvalue = 4.66, % of Variance = 17.63%), Isolation and overidentification (Items 13, 18, 20, 24, 25, Eigenvalue = 3.03, % of Variance = 14.77%), Self-judgement (Items 1, 8, 11, 21, Eigenvalue = 1.33, % of Variance = 11.40%), and Common humanity (Items 3, 7, 10, Eigenvalue = 1.20, % of Variance = 9.96%). The model structure was tested with confirmatory factor analysis and the fit to the data was found to be good, χ^2^/139 = 2.141, *p* < .005, CFI = .931, GFI = .956, SRMR = .057, CI 90% [0.044, 0.061], RMSEA = .053. The reliability of the subscales ranged from α = .70 to α = .81.

#### Almost Perfect Scale Revised (APS-R)

To measure perfectionism, the Almost Perfect Scale (APS-R, Slaney et al., 2001) translated and adapted in Greek by Diamantopoulou and Platsidou (2014) was used. It comprises 23 items, of which 7 items refer to High Standards (e.g., *I expect the best from myself*), 12 items assess the Discrepancy (e.g., *Doing my best never seems to be enough*), and 4 items refer to Order (e.g., *Neatness is important to me*). Participants are asked to evaluate their agreement with each item using a 5-point Likert scale ranging from 1 (I strongly disagree) to 5 (I strongly agree). The scale classifies participants into different types of perfectionists (adaptive, maladaptive, non-perfectionists) by taking into consideration their scores on High Standards and Discrepancy subscales. The Order subscale is not included in the classification as it is considered a neutral and not a core dimension of perfectionism (Stoeber & Otto, 2006).

In the present study, an exploratory factor analysis of the APS-R proceeded which revealed a three-factor solution that perfectly matched the inner structure of the original scale and explained the 59.35% of the total variance (High Standards explained the 32.11% of the total variance with eigenvalue 7.14, Discrepancy explained the 15.16% of the total variance with eigenvalue 4.4, and Order explained the 12.08% of the total variance with eigenvalue 1.53). The model structure was tested with confirmatory factor analysis and the fit to the data was found to be good, χ^2^/200 = 2.705, *p* < .005, CFI = .930, GFI = .928, SRMR = .062, CI 90% [0.058, 0.071], RMSEA = .064. The reliability of the subscales ranged from α = .79 to α = .93.

### Procedure

Participants were recruited for the study via e-mail, using the Google Forms web application and via social media applications. The participants were asked to answer demographic questions before completing the basic self-reported questionnaires. The study was in line with rules of ethics of the American Psychological Association and with the European Union Regulation on sensitive personal data. As far as procedure is concerned, during the data collection the anonymity of the respondents was ensured. Participants were adults and consented to voluntarily participate in the study as well as to the publication of the results. The data were collected within one month of data collection during January 2023.

### Data Analysis

Initially, a hierarchical method of clustering was performed to help identify groups of participants with similar characteristics of perfectionism. The cluster centroids using the Ward density method based on the Euclidean distance between cases, followed by a non-hierarchical K-means cluster analysis yielded three clusters. Taking into consideration that there is not an unanimously accepted statistical method to determine the clusters, their number was defined by a) the distance between cluster steroids, b) the number of participants in each of them, and c) the statically significant differences indicated by one-way analysis of variance (Sugar & James, 2003). Three clusters were identified which corresponded to the three different perfectionistic types (adaptive, maladaptive, non-perfectionists) presented in the results.

Before checking for the hypotheses, preliminary descriptive and intercorrelation analyses (means, standard deviations, skewness, and kurtosis values, and correlations) using the statistical package SPSS (Version 26) were performed to determine the bilateral relationships between perfectionism and self-compassion and to ascertain whether the parametric analyses could be applied on the data. To test Hypothesis 1a and 1b, correlation and regression analyses were performed to examine the association between the two constructs and the predictive strength of the perfectionism dimensions (High Standards, Discrepancy) on the positive and negative self-compassion components. To test Hypothesis 2a and 2b, a series of Univariate Analyses of Variance (ANOVA) were applied to check if the three types of perfectionists emerged demonstrate differences related to the self-compassion components. Prior to checking the last hypothesis regarding the moderating effect of age in the relation between the types of perfectionism and the self-compassion components, Univariate Analyses of Variance (ANOVA) were applied to check for age differences in the perfectionistic types and the self-compassion components. Finally, the moderating effect of age in the relationship of perfectionistic types to self-compassion components was checked, using SPSS PROCESS (Version 3.5, Model 1, 95% confidence interval, 5.000 bootstrap samples).

## Results

### Types of Perfectionists

Initially, a hierarchical method of clustering was performed to help identify groups of participants with similar characteristics of perfectionism. The cluster centroids using the Ward density method based on the Euclidean distance between cases, followed by a non-hierarchical K-means cluster analysis yielded that three or four cluster solutions were more suitable. The preferred solution was that of three clusters, which also complies with the relevant literature. Table 1 presents the final cluster centroids. Cluster 1 (*N* = 197) includes those participants who reported the lowest levels of high standards and, thus, it represents the type of non-perfectionists. Cluster 2 (*N* = 135) involves the participants who scored high in both high standards and discrepancy describing the maladaptive type of perfectionists. Finally, Cluster 3 (*Ν* = 177) incorporates those who scored high on high standards but low on discrepancy representing the adaptive type of perfectionists.

**Table 1:** Final Cluster Centers (Means)

	Cluster 1 (*N* = 197)	Cluster 2 (*N* = 135)	Cluster 3 (*N* = 177)	
Variable	Non-perfectionists	Maladaptive perfectionists	Adaptive perfectionists	*F* (2,506)
High Standards	3.28	4.26	4.40	419.09**
Discrepancy	2.45	3.70	2.17	465.46**

**p < .001.

### The Predictive Role of Perfectionistic Dimensions to Self-Compassion Components

Before checking for the hypotheses, means, standard deviations, and skewness and kurtosis values were estimated (see Table 2). Results indicated that participants reported higher levels in the positive self-compassion components and the positive perfectionism dimension compared to the negative self-compassion components and the maladaptive perfectionism dimension. In addition, it was found that skewness values ranged from -0.84 to 0.56 and kurtosis values ranged from -0.78 to 0.22. At the next step, the correlations between the self-compassion components and the perfectionism dimensions were estimated. The results showed that the High standards dimension was positively correlated with both the positive and negative components of self-compassion, while the Discrepancy dimension was found to negatively correlate with the positive components of self-compassion and positively correlate with the negative components of self-compassion.

**Table 2:** Means (and Standard Deviations) and Correlations Between Self-Compassion and Perfectionism

				SCS				APS	
Variable	*M* (*SD*)	Skewness	Kurtosis	1	2	3	4	5	6
1. SCS Self-Kindness and Mindfulness	3.22 (0.74)	-0.12	-0.09						
2. SCS Isolation and Overidentification	2.77 (0.89)	0.17	-0.66	-.29**					
3. SCS Self-Judgment	2.92 (0.84)	0.08	-0.49	-.15**	.49**				
4. SCS Common Humanity	2.90 (0.92)	0.06	-0.78	.49**	-.09*	.03			
5. APS High Standards	3.39 (0.66)	-0.29	-0.55	.14**	.20**	.25**	.01		
6. APS Discrepancy	2.68 (0.77)	0.56	-0.09	-.32**	.51**	.46**	-.12**	.16**	
7. APS Order	4.15 (0.72)	-0.84	0.22	.22**	-.03	.08	.12**	.45**	.01

*p < .05. **p < .001.

For testing Hypotheses 1a (Discrepancy will negatively predict the positive self-compassion components and positively predict the negative self-compassion components) and 1b (High Standards will not predict any of the self-compassion components), a series of regression analyses were performed. The results, presented in Table 3 confirmed Hypothesis 1a, since Discrepancy was found to positively predict Self-judgment and Isolation and overidentification and negatively predict Self-Kindness and mindfulness and Common humanity. However, Hypothesis 1b was not confirmed. The results showed that High Standards positively predicted Self-judgment, Self-Kindness and mindfulness, and Isolation and overidentification. It should be noted that the predictive effect of Discrepancy on self-compassion components was greater compared to the effect of High Standards. The Order dimension of perfectionism was not found to predict any of the self-compassion components.

**Table 3:** Regression Analyses of Perfectionism Predicting Self-Compassion

Predictor	*F*(4, 504)	*R*	*R^2^*	*p*	β	*SE*	*t*
**Model 1: Self-Kindness and Mindfulness**	22.282	.387	.150	< .001			
High Standards				< .001	.24	.05	5.03
Discrepancy				< .001	-.32	.04	-8.05
**Model 2: Self-Judgment**	40.095	.491	.241	< .001			
High Standards				< .001	.23	.05	4.41
Discrepancy				< .001	.47	.04	10.94
**Model 3: Isolation and Overidentification**	61.728	.573	.323	< .001			
High Standards				.005	.14	.05	2.80
Discrepancy				< .001	.56	.04	13.02
**Model 4: Common Humanity**	5.613	.169	.207	< .001			
High Standards				.187	.08	.06	1.32
Discrepancy				.011	-.14	.05	-2.55

### Differences of Self-Compassion Components in Relation to Perfectionistic Types

At the next step, a series of Univariate Analyses of Variance (ANOVA) were applied to check Hypotheses 2a and 2b and to examine whether there were differences on the self-compassion components between the perfectionistic types (adaptive, maladaptive, non-perfectionists). The findings presented in Table 4 indicated that adaptive perfectionists and non-perfectionists reported higher levels on the positive components of self-compassion and lower levels on the negative components of self-compassion compared to maladaptive perfectionists, while maladaptive perfectionists reported higher levels on the negative components of self-compassion and lower levels on the positive components of self-compassion compared to adaptive and non-perfectionists, and, thus, Hypotheses 2a and 2b were partially confirmed, since no statistically significant differences were found concerning the Common humanity component of self-compassion.

**Table 4:** Differences in Self-Compassion Components in Relation to Perfectionistic Types

Self-Compassion Components/Types of Perfectionists	*Μ*	*SD*	Significant differences between groups	*F*(2, 506)	*p*
SCS—Self-Kindness and Mindfulness				19.39	< .001
Adaptive perfectionists (AP)	3.47	0.75	AP-NP		< .001
Maladaptive perfectionists (MP)	2.97	0.74	AP-MP		< .001
Non-perfectionists (NP)	3.16	0.66	MP-NP		.049
SCS—Isolation and Overidentification				56.88	< .001
Adaptive perfectionists (AP)	2.55	0.82	AP-MP		< .001
Maladaptive perfectionists (MP)	3.41	0.77	MP-NP		< .001
Non-perfectionists (NP)	2.53	0.83			
SCS—Self-Judgement				47.59	< .001
Adaptive perfectionists (AP)	2.80	0.86	AP-MP		< .001
Maladaptive perfectionists (MP)	3.47	0.73	MP-NP		< .001
Non-perfectionists (NP)	2.65	0.72			
SCS—Common Humanity				2.57	.078

### Differences in Self-Compassion and Perfectionism in Relation to Age

At the next step, participants were divided into age groups that covered emerging adulthood (18–29 years, *N* = 178, *M* = 24.65, *SD* = 3.53), established adulthood (30–45 years, *N* = 167, *M* = 37.99, *SD* = 4.23), and middle adulthood (46–65 years, *N* = 164, *M* = 57.06, *SD* = 7.05), following the proposed age taxonomy suggested by many researchers (e.g., Mehta et al., 2020; Reifman & Niehuis, 2023). Since the relevant literature has identified age as a demographic variable associated with both perfectionism and self-compassion, along with the fact that studies addressing to middle-aged and older adults are so far rather limited, splitting the sample into groups could help identify differences over time on the dimensions of perfectionism and/or the self-compassion components.

As Table 5 shows, in general, participants covering established and middle adulthood reported higher levels on the positive self-compassion components and lower levels on the negative self-compassion components compared to participants covering emerging adulthood. Contrariwise, emerging adults scored higher in perfectionism dimensions compared to established and middle adults. Thus, Hypothesis 3a was confirmed.

**Table 5:** Differences in Self-Compassion and Perfectionism in Relation to Age

Variable/Age groups	*Μ*	*SD*	Significant differences between groups	*F*(2, 506)	*p*
SCS—Self-Kindness and Mindfulness				3.79	.023
Emerged adulthood (18–29 years) (1)	3.05	0.75	1–2		.050
Established adulthood (30–45 years) (2)	3.27	0.78	1–3		.030
Middle adulthood (46–65 years) (3)	3.28	0.71			
SCS—Isolation and Overidentification				13.43	< .001
Emerged adulthood (18–29 years) (1)	3.12	0.88	1–2		.002
Established adulthood (30–45 years) (2)	2.75	0.97	1–3		< .001
Middle adulthood (46–65 years) (3)	2.61	0.82			
SCS—Self-Judgment				7.61	< .001
Emerged adulthood (18–29 years) (1)	3.21	0.85	1–2		< .001
Established adulthood (30–45 years) (2)	2.89	0.84	1–3		.003
Middle adulthood (46–65 years) (3)	2.72	0.90			
SCS—Common Humanity				5.47	.002
Emerged adulthood (18–29 years) (1)	2.63	0.94	1–2		.050
Established adulthood (30–45 years) (2)	2.91	0.92	1–3		.001
Middle adulthood (46–65 years) (3)	3.01	0.89			
APS—High Standards				11.59	< .001
Emerged adulthood (18–29 years) (1)	4.16	0.62	1–2		.049
Established adulthood (30–45 years) (2)	3.96	0.68	1–3		< .001
Middle adulthood (46–65 years) (3)	2.81	0.63			
APS—Discrepancy				4.59	.011
Emerged adulthood (18–29 years) (1)	2.87	0.83	1–2		.021
Established adulthood (30–45 years) (2)	2.63	0.80	1–3		.019
Middle adulthood (46–65 years) (3)	2.61	0.71			

### The Moderating Role of Age on the Relation Between Perfectionism and Self-Compassion

To check Hypothesis 3b, a series of moderation analyses where the independent variables were the types of perfectionism, the dependent variables were the self-compassion components and the moderator was the age-groups was performed, in order to check if the effect of the different perfectionistic types on self-compassion components varies as a result of proceeding age. Four models were checked it total in which the age groups moderated the relationship between the perfectionistic types and the self-compassion components: Self-Kindness and Mindfulness (Model 1), Isolation and Overidentification (Model 2), Self-Judgement (Model 3), and Common Humanity (Model 4). Results partially confirmed Hypothesis 3b, since only one statistically significant model was found.

**Figure 1:**
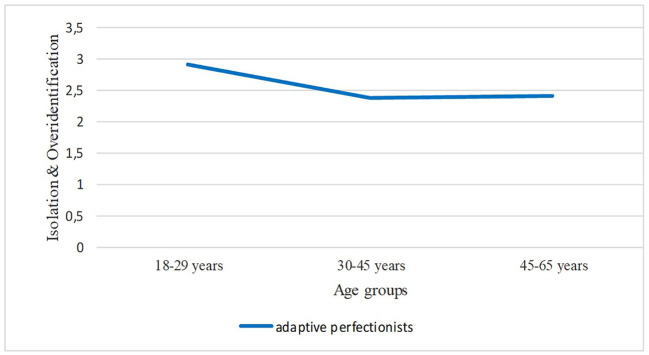
Moderating Effect of Age on the Relation Between Adaptive Perfectionistic Type and Isolation and Overidentification

More specifically, results indicated a statistically significant model (*R* = .48, *R^2^* = .23, *p* < .001) in which a significant moderation effect for the interaction of perfectionistic types and age on Isolation and overidentification, β = -.64, *SE* = .24, *t* = -2.63, *p* = .008, 95% CI [-1.12, -0.16], was observed. Analysis of the moderation effect indicated that the interaction effect was significant only for adaptive perfectionists, β = -.38, *SE* = .15, *t* = -2.54, *p* = .002, 95% CI [-0.68, -0.09], (see Figure 1).

## Discussion

This study sought to broaden our understanding on the relationships between two multidimensional constructs, self-compassion and perfectionism, and to highlight their associations with age. Some interesting findings were obtained.

To begin with, Hypothesis 1a of the study aimed at checking if the maladaptive dimension of perfectionism negatively predicts the positive self-compassion components and positively predicts the negative self-compassion components. The present study replicates the findings of previous studies (e.g., Linnett & Kibowski, 2020) in finding that Discrepancy positively predicted Self-Judgment and Isolation and Overidentification and negatively predicted Self-Kindness and Mindfulness and Common Humanity. The distress resulting from not being able to respond to unrealistic high self-imposed standards entails to harsh self-criticism and self-judgement (Linnett & Kibowski, 2017). It seems that there is a strong link between how people judge themselves based on their ability versus their actual performance; those who experience high discrepancies may be less compassionate towards themselves than those whose expectations align better with what they actually achieve. This presence of difference between expected and actual performance could lead to lower self-compassion levels, as people may find it challenging to extend kindness towards themselves and/or may internalize negative self-judgments and perceive compassion towards oneself as undeserved or ineffective (Robinson et al., 2016).

In addition, it was hypothesized that the adaptive perfectionism dimension (High Standards) will not predict any of the self-compassion components (Hypothesis 1b), since studies on the relation between the two constructs highlight the complexity of the relationship between the adaptive perfectionism dimensions and the self-compassion components suggesting that there is either a weak negative association between the adaptive perfectionism dimensions (e.g., striving for excellence, perfectionistic strivings) and the negative self-compassion components namely Self-judgement (e.g., Hiçdurmaz & Aydin, 2017; Linnett & Kibowski, 2020), or that there is no association between them (e.g., Neff, 2003a). However, in the present study, it was found that the High Standards dimension positively predicted the negative self-compassion components (Self-judgment and Isolation and Overidentification). This empirical observation may find its underpinning in a theoretical framework suggesting that individuals with high standards tend to engage in self-judgment when facing setbacks, possibly due to their perception of these failures as deviations from their exacting criteria. High standards can also lead to social isolation, as individuals fear not meeting their lofty expectations, making them hesitant to seek external support. In addition, those with high standards may excessively identify with their failures, driven by their significant investment of self-worth in achieving success, which can result in a profound immersion in negative thoughts and emotions known as overidentification.

Interestingly, it was also found that High Standards positively predicted Mindfulness and Self-Kindness, a finding that is unique in the already existing literature. While many may view high standards as a stressful burden on mental health, recent studies indicate the opposite; that having high standards may actually be beneficial for well-being and personal growth since, instead of being discouraged by failure, those with perfectionistic tendencies view their shortcomings as an opportunity for improvement (Dunkley et al., 2003; Suh & Shiqin Chong, 2022). Individuals who set high standards for themselves are more likely to engage in mindfulness and self-kindness practices that lead toward an improved sense of well-being and personal growth (Suh & Shiqin Chong, 2022).

Finally, it should be noted that the predictive effect of Discrepancy on self-compassion components was stronger compared to the effect of High Standards, a finding that is consistent in previous studies (e.g., Hiçdurmaz & Aydin, 2017; Mosewich et al., 2011). The negative impact of perceived failure may be a potential explanation for this finding. This implies that individuals are less likely to experience feelings of self-compassion towards themselves when they perceive a discrepancy between their current state and their desired state, as opposed to simply holding high standards for themselves, since they may experience guilt or shame—emotions that have been linked with lower levels of self-compassion (Sirois et al., 2019; Woods & Proeve, 2014). This finding can have several implications. Firstly, it suggests that interventions aimed at increasing self-compassion may be more effective if they focus on helping individuals recognize discrepancies between their current selves and ideal selves, rather than solely promoting the adoption of high standards. Additionally, it highlights the importance of addressing negative emotions surrounding perceived failures or shortcomings in order to cultivate greater levels of self-acceptance.

With regards to Hypotheses 2a and 2b, it was expected that the perfectionistic types would present differences on the self-compassion components. The findings of the study indicated that adaptive perfectionists and non-perfectionists reported higher levels on the positive and lower levels on the negative components of self-compassion, while maladaptive perfectionists reported higher levels on the negative components of self-compassion and lower levels on the positive components of self-compassion. The multidimensional approach of self-compassion in this study contributes to a more detailed understanding of their relationships, instead of simply approaching self-compassion as a positive attitude and perfectionism as a negative trait.

To begin with, as it has already been suggested by the literature, maladaptive perfectionism is negatively associated to self-compassion (Ferrari et al., 2018; Stoeber, Madigan, & Gonidis, 2020; Yeshua et al., 2019). Findings reporting low levels of positive self-compassion components and high levels of negative self-compassion components in maladaptive perfectionists may be related to and explained by the irrational cognitions associated with the constant desire of being perfect and the intolerance towards flaws and imperfections (Abdollahi et al., 2020; Ferrari et al., 2018). According to Kawamoto and his colleagues (2023), individuals dealing with maladaptive perfectionism hold unattainable desires for flawlessness and exceptionally lofty standards that make them hypercritical about themselves. This conduct could be due to their inclination towards self-validation instead of internalizing self-compassion (Tobin & Dunkley, 2021).

On the other hand, research suggests that adaptive perfectionists tend to be more self-compassionate and have better mental health outcomes compared to maladaptive perfectionists (Sirois & Molnar, 2016; Stoeber & Otto, 2006). Studies have found that adaptive perfectionists tend to report higher levels on positive self-compassion components than maladaptive perfectionists, because they are able to acknowledge their imperfections without harsh self-criticism and are more likely to practice self-care (Stoeber & Damian, 2014; Stoeber & Otto, 2006). In addition, they demonstrate mindfulness and kindness towards themselves to manage the stress associated with striving for excellence (Kawamoto et al., 2023). It seems that, by cultivating a sense of self-compassion and accepting one's own imperfections alongside striving for excellence, individuals can achieve higher levels of well-being while avoiding the negative effects often seen in non-adaptive forms of perfectionism, since the challenges are faced as an opportunity for growth and improvement (Dunkley et al., 2003).

Finally, studies consistently show that non-perfectionists have an easier time accepting their imperfections than those who strive for perfectionism. Non-perfectionists tend to have a more accepting and forgiving attitude towards their performance and may not place as much emphasis on achieving high standards or avoiding shortcomings. Stoeber, Lalova, and Lumley (2020), found that non-perfectionists exhibit more self-kindness when confronted with personal failure or perceived inadequacy. Thus, they may be more likely to recognize that imperfection is a normal part of the human experience and that mistakes do not define their self-worth (Neff & McGehee, 2010). By acknowledging their limitations without judgement or blame, non-perfectionists develop a stronger sense of worthiness which is translated into greater psychological well-being (Stoeber, Lalova, & Lumley, 2020).

It is important to note that self-compassion and perfectionism are dynamic and multidimensional constructs that can vary across situations and contexts and can be influenced by various factors such as personality, culture, and life-experiences. Therefore, their levels may vary depending on individual differences and situational factors. For this reason, differences between the participants in self-compassion components and types of perfectionism in relation to their age were examined (Hypothesis 3a). The findings showed that, overall, participants in established and middle adulthood reported higher levels on the positive self-compassion components and lower levels on the negative self-compassion components compared to the participants covering emerging adulthood. On the opposite direction, emerging adults scored higher in both the adaptive and maladaptive perfectionism dimensions (Discrepancy and High Standards).

The differentiating role of age in self-compassion levels is often reported in the Greek and international literature (e.g., Homan, 2016; Karakasidou et al., 2020; Neff & Pommier, 2013). Neff (2011) suggests that it may be possible that individuals adopt more self-compassionate responses towards their failures and/or imperfections as they grow older. From a developmental point of view, self-compassion is expected to increase across lifespan, taking into consideration the physical, cognitive, and emotional changes that occur over time. Behavioral and psychophysiological studies suggest that adolescence is a challenging developmental period characterized by insecurity about self-image, struggling with managing emotions and/or manifestation of risky behaviors (Pfeifer et al., 2011). In contrast, people over 65 years are positioned on the last stage in Erikson’s theory (1968) which involves the accomplishment of integrity. Integrity refers to one’s ability to reflect back in their life with a sense of fulfillment and involves, inter alia, a sense of wholeness, acceptance and lack of regret (Erikson, 1968). The exposure to stressful life events enhances resilience, which in turn allows for a gentler attitude towards oneself. The maturity that comes with age favors a cognitive and empathetic understanding, that may increase the sense of interconnectedness when dealing with difficulties (Karakasidou et al., 2020; Neff, 2011).

The exact relationship between age and perfectionism remains unclear and studies have reported mixed findings. However, there are studies suggesting that younger individuals display higher levels of maladaptive perfectionism, while older individuals exhibit more adaptive forms of perfectionism (Curran & Hill, 2019; Sand et al., 2021). This could be attributed to the fact that younger adults are still navigating their way through the challenges associated with emerging adulthood, such as transitioning from school to work or developing new relationships. During this period, they may feel increased pressure to succeed in all aspects of life, leading them to set unrealistic standards for themselves which can contribute to maladaptive perfectionist tendencies. Curran and Hill (2019), in a meta-analysis of 164 different samples, found that the perfectionistic concerns of emerging adults have been increased compared to those of previous generations, a fact attributed to cultural changes. In established adulthood, individuals still have high standards since most of them are preoccupied with advancing their careers while also taking on the duties of an intimate partner and raising children (Mehta et al., 2020). These findings explain why emerging and established adulthood are more often associated with higher levels of maladaptive perfectionism compared to middle adulthood. Robinson and his colleagues (2021) suggested that middle-aged adults still have goals to strive for, but this does not necessarily coincide with perfectionism. However, further research is required to clarify whether the age differences reporting in studies emerge as a consequence of generational dissimilarities or by perfectionism being a trait characteristic that can be modified through lifespan.

The last hypothesis of the study (Hypothesis 3b; age would moderate the relationship between the perfectionistic types and the self-compassion components) concerned the examination of the effect of age on the relationship between the types of perfectionism and the self-compassion components. Interestingly, results did not confirm our hypothesis, since all the moderating effects were found non-significant. The only statistically significant moderating effect of age found was on the relationship between adaptive perfectionism and isolation and overidentification. It is possible that younger adaptive perfectionists are more likely to experience Isolation and overidentification than older individuals due to differences in life experiences and coping mechanisms. This finding is supported by Curran and Hill (2019), who found that older individuals with high levels of adaptive perfectionism were less likely to feel isolated than their younger counterparts. Overall, the results of the present study suggest that the relation between the two constructs is relatively constant and does not depend on the effect of age. This finding could be taken into consideration when designing interventions including perfectionism to enhance self-compassion. It is possible, however, that there are other demographic factors (e.g., gender, ethnicity) and/or other variables (e.g., resilience) that may moderate this relationship.

### Limitations, Future Directions, and Contribution

With regard to limitations on sampling method, self-report measures were used to collect the data. The weaknesses of such tools (e.g., subjectivity, social desirability bias) are often discussed in behavioral research (Brutus et al., 2013). In addition, the questionnaires were distributed electronically. Thus, a convenience sample was recruited and, given the educational level of the participants, it is possible that only highly educated and/or motivated adults participated in the study.

Finally, the results of the study were emerged from cross-sectional data and, thus, associations between self-compassion and perfectionism were reported, but no causative relationships. Future longitudinal studies can overcome these limitations with repeated observations on the examined variables. As the discussion on self-compassion and perfectionism continues, future research should aim to provide explanations on the developmental trajectory of the two constructs and it is important to continue examining whether demographical and cultural factors generate differences between samples and/or moderate the relationship between the two constructs.

To sum up, the present study contributes to the fast-growing body of literature that examines the association between multidimensional perfectionism and self-compassion. It is important to understand that there are different types of perfectionism, namely adaptive and maladaptive perfectionism, each with distinct outcomes, especially on the self-compassion components—it is reminded that High Standards dimension positively predicted Mindfulness and Self-Kindness. In addition, results suggest that the different types of perfectionism differ in relation to self-compassion components, a finding that could have practical implications. Specifically, cultivating self-compassion may be an effective strategy for individuals who struggle with maladaptive perfectionism and its associated negative outcomes. Incorporating self-compassionate practices into daily life could help individuals develop a more balanced perspective on success and failure. Overall, embracing self-compassion as a complementary approach to addressing perfectionistic tendencies may prove beneficial for those seeking greater resilience and overall well-being.
